# Community Health Workers’ experiences of a package providing increased support and supervision - a qualitative study of a home visiting model in rural South Africa

**DOI:** 10.21203/rs.3.rs-3333610/v1

**Published:** 2023-09-27

**Authors:** Linnea Stansert Katzen, Sarah Skeen, Elaine Dippenaar, Christina Laurenzi, Vuyolwethu Notholi, Karl le Roux, Ingrid le Roux, Ncumisa WaluWalu, Nokwanele Mbewu, Mary Jane Rotheram Borus, Mark Tomlinson

**Affiliations:** Stellenbosch University; Stellenbosch University; Stellenbosch University; Stellenbosch University; Stellenbosch University; Stellenbosch University; Philani Maternal, Child Health and Nutrition Trust; Zithulele Research Center; Philani Maternal, Child Health and Nutrition Trust; University of California; Stellenbosch University

**Keywords:** Community Health Workers, Supervision, Home visiting, Training, Resources

## Abstract

Deploying Community Health Workers is a crucial strategy to improve health at a community level in low and middle income countries. While there is substantial evidence for CHW effectiveness, there is a need for more research on the mechanisms through which these programs work. Understanding CHWs experiences of how programmes function is important. This article examines CHW’s experiences of three key programmatic domains; training, logistical support and supervision. Data were gathered using a qualitative study embedded within a cluster randomized controlled trial of an enhanced supervision package delivered to government-employed CHWs in the rural Eastern Cape, South Africa.

We interviewed CHWs (n = 16) and two supervisors. Three overarching areas and five sub-themes emerged from our interviews. CHW knowledge and confidence increased through additional training, that CHW motivation and community acceptance improved because of added logistical support, and that CHW supervision led to improved sense of accountability, feelings of respect, and sense of being supported.

Our findings highlight the importance of a functional support system within which CHWs can operate, in a context where most CHWs operate in isolation and without support. CHWs receiving supportive supervision reported positive impacts on their motivation and ability to carry out their work effectively.

## Introduction

Deploying Community Health Workers (CHWs) is an important strategy to improve access to health care and to improve population health in low and middle income countries (LMICs) [1, 2]. While there is substantial evidence that CHW programs improve a range of health outcomes [1, 3–5], these benefits tend to reduce or disappear when CHW programs are scaled up. In addition, there is limited research that has successfully identified the mechanisms through which these programs work and how work place-related factors, such as supportive work environments for CHWs, impact on outcomes [2, 6]. This article describes how CHWs experience three programmatic domains (training, logistical support and supervision), identified by CHWs in this study and others [3] as key for supportive work environments, in a rurally-based CHW programme in South Africa.

### Training.

The quality and quantity of training of CHWs varies significantly across programs, but is often reported as insufficient or of low quality [3, 7]. There is evidence that higher quality training and follow up training improves CHW knowledge substantially [8]. The training however needs to be up to date and be responsive to current health needs [9].

### Logistical support.

Improving access to equipment and provision of logistical support such as transport has been shown to improve CHW motivation and performance [10, 11]. CHWs often report shortfalls in equipment and logistical support provided, which poses significant challenges to successful implementation of programs [3]. CHWs often work in rural and remote areas and access to transport and basic equipment is key to effective delivery [3].

### Supervision.

Supervision is often described as the “missing element” that ensures the efficiency of CHW programs – particularly when programs are scaled up [3, 12, 13]. Despite extensive evidence about the importance of supervision for CHW program success [2, 14–15], supervision processes are often reported as absent, or of poor quality [16, 17].

Similar to in many other countries, South Africa has a national, government-funded CHW program [18]. In South Africa, CHWs are part of Ward Based Outreach Teams [WBOTs] [19]. These teams work at a ward level (the political sub-division of a municipality), where a group of six to ten CHWs conduct home visits and provide basic information about non-communicable diseases, HIV/TB treatment, and maternal and child health. CHWs in the WBOT system are supervised by primary health care clinic managers and operational team leaders [18, 19, 20]. Although CHWs have described feeling positive about their jobs [21], and the WBOT strategy is promising in its design [22] - challenges have been reported relating to the programmatic domains described above; training, logistical support and supervision. It is clear that the South African CHW expansion is only likely to be successful if investments are made in these areas [2, 23–25].

Considering the domains of the supportive system outlined above, an intervention package including in-the-field training, logistical support and supervision – based on the Philani Mentor Mother model (described below) was developed. This intervention was implemented to see whether CHW program outcomes improve when these domains are provided. We hypothesize that additional training, logistical support and supervision, will help CHWs to more easily perform their work and improve their job satisfaction.

## Methodology

This study is a qualitative descriptive study drawing from semi-structured interviews with community health workers enrolled in the intervention and control arms of an cRCT called the Eastern Cape Supervision Study (ECSS). The objective of the ECSS was to investigate whether good quality supervision and support provided to South African government CHWs (intervention) improved maternal and child outcomes when compared to routine supervision (control) as delivered within the primary health care system. The cRCT has recently been completed and the results are being analysed. The study protocol details all processes [26].

## Setting

The study was conducted in the rural Eastern Cape province of South Africa. The study district is one of the most under-developed and impoverished municipalities in the country. Access to healthcare is a challenge in this area, and employment levels are low [27, 28]. Previous research in the area reported that 5% of mothers have never attended school, only 6.6% had a high school diploma and 92.5% of households received some kind of government grant. Fewer than 50% of children had up-to-date immunisations by three months, and at 12 months, 73.1% had their immunisations up to date [29]. Health care is mainly provided by a government district hospital and surrounding primary care clinics, though there are a few private health care practitioners in the area. **HWs**

## Ethics

This study, was approved by the Stellenbosch University Health Research Ethics Board (N16/05/064), by the UCLA Institutional Review Board (IRB#16–001362) and by the Eastern Cape Department of Health.

## CHWs

For the cRCT, 37 CHWs from eight clinics were recruited.

### Training.

CHWs in both control and intervention conditions were provided with additional general CHW training. Training included four weeks of group-based training session followed by two weeks of mentoring by experienced CHWs in the field. The training included modules such as: counseling and communication skills, negotiating entry, HIV, TB, peri-natal health, infant feeding and nutrition, and self-care [30, 31]. Once training was completed, CHWs in the intervention condition took part in an enhanced supervision intervention, where supervisors provided support and supervision for each CHW biweekly at a minimum. CHWs in the control condition continued to work in the standard Department of Health system, supervised by existing Operational Managers and Team Leaders based at the clinics.

### Logistical support.

Intervention CHWs had access to transport at least twice a month when seen by a supervisor in the field. CHWs in the control arm did at the time of the study not have access to any mode of transport. Intervention CHWs also received additional equipment and medication such as folders, scales, thermometers, de-worming medication and vitamin A. This added support and equipment were not available for CHWs in the control condition.

### Supervision.

CHWs in the intervention condition received support and supervision from Philani supervisors. Two Philani supervisors were recruited to provide supportive supervision to 10 CHWs each in the intervention condition. Previous evaluations of this approach have shown a positive impact on maternal and child health [4, 31]. CHWs in the control condition continued to work in the standard Department of Health system, supervised by existing Operational Managers and Team Leaders based at the clinics.

## Sample

We interviewed eight CHWs from each arm of the cRCT, and two supervisors from the intervention arm individually (total n = 18). CHWs from each of the study clinics were randomly drawn from a hat and approached for interviews. At this point, content saturation was reached.

## Data collection

We conducted interviews three months’ post-last follow up in the trial, in June and July 2021. Interviews were conducted in a private space at a local training and research centre. Informed, voluntary consent was obtained before any data were collected. It was made clear to all participants that they were not required to participate in the study. Given the small pool of participants, and that all CHWs were employed by the Eastern Cape Department of Health (DoH), extra consideration was given to issues of confidentiality. During the informed consent process, CHWs were assured that their interviews would be de-identified and anonymized, and that information given would in no way affect their work as CHWs. Interviews were conducted in isiXhosa by a trained data collector with extensive training and experience of conducting in-depth qualitative interviews. She was not known to the participants. Interviews were translated to English and transcribed by a separate team at Stellenbosch University who received de-identified audio recordings, and transcripts were then sent to the research team. Participant identification numbers (PIDs) were used to de-identify participants. In addition, names of clinics and references to geographical information (including distance) or other identifying information was removed from transcripts to further anonymise the data. Anonymity was more difficult to enforce with supervisor participants, given that there were only two participating. We addressed this through removing identifying information from any data files. As the supervisors were not employed by the DoH, we assessed the risk of identification as less serious than for the DoH employed CHWs. Furthermore, we shared the quotes and draft article with the supervisors and they agreed to have it published.

## Data analysis

Thematic analysis was used to analyse these data, structured by the six steps described by Braun and Clarke [2006]: familiarization, generating initial codes, searching for themes, reviewing themes, defining and naming themes, and producing the report [32]. Transcribed interviews were reviewed line by line, and a preliminary coding scheme was developed. This coding scheme was then presented to members of the team to validate and discuss the identified themes [33]. ATLAS.ti software was used for coding, naming, and organizing data. Once theme saturation had occurred, data extracts from each transcript were grouped together under each category [34]. In order to ensure objectivity and validate the analysis of the data, a second researcher (CL) analyzed randomly-selected sections of data, and discrepancies were resolved through discussion [34]. Codes were collapsed into code groups and themes were derived.

## Results

Of the 16 CHWs in this study, 15 were female and one was male. They had all been working as CHWs for varying periods of time prior to the implementation of this study, ranging from 13 months to 24 years. Ages ranged between 39 and 59 years.

Three overarching areas and five sub-themes emerged from our interviews

### 1. CHW knowledge and confidence

#### 1.1 Additional training leads to increased CHW knowledge and confidence

Respondents described the importance of the increased knowledge they received through the training provided to CHWs.

*“It [the training] had an influence in the way I worked. When I came back from [the initial CHW training] I did not know certain things. But when I came back from [the Philani] training I knew a lot of things” (*Control CHW)

*“I speak about things with power now. I don’t just say anything I’m not sure of. I now know that if I’m headed to a specific household, these are things I’m going to say”* (Intervention CHW)

The improved knowledge also appears to have improved the CHWs credibility in the community, while the improved service delivery also created increased expectations from community members, which in turn appeared to motivate CHWs

*“There’s a huge difference sis, because that time we were not trained by Philani we were getting trainings but not with Philani; the Philani training made a huge difference in my work experience because it had materials; we were trained and received the materials, you get trained then you also do what you were trained for, and clients notice that there’s a huge difference. This and that wasn’t happening before and when it’s a visit time you see that everyone is excited; that’s what gave us higher level. They see that the nurses have arrived, you will see other people arriving from other houses, the neighbours will come because they see we are working here”* (Intervention CHW*)*

### 2. CHW motivation and community acceptance

#### 2.1 Improved community acceptance and program credibility with logistical support

What also emerged strongly was the effect that the added equipment had on program credibility within the community. CHWs in the intervention arm described a sense that they were able to deliver improved services as a result. Intervention CHWs indicated that resources - especially equipment such as scales, backpacks, folders, medication and phones, substantially improved their ability to do their work. Having access to equipment was reported to be strongly linked to acceptance of the intervention by households in the community, and the credibility of the program. CHWs reported that they felt like they had something “real” to contribute with, as they could bring this equipment and new knowledge into the community and assist community members. The scales in particular appear to have improved household acceptance and served as an entry point when visiting households.

*“We were given scales by Philani and that made us feel important because for people in rural areas to have that is a big deal for them” ([*Intervention CHW)

*“Having transport and all the necessary machines like BP [blood pressure] machines and scales because it gives us dignity and respect from the community. They know that we are working. Other patients would invite us to monitor them even if it’s not their appointment date just because they know we have the right and helpful equipment. This equipment is important in the rural areas because they are not always available.” (*Intervention CHW)

*“At least now you can get on a scale because before we were just carrying our book into a plastic, take a pen and that was all, but now you know that you are going to work, you take your backpack put in your cotton wool, jars, forms, folder and cell phone and when you arrive to a house for … It made us people that means wow.” (*Intervention CHW)

The fact that the intervention group had access to a car when being visited by a supervisor in the field made a big difference and helped CHW get to difficult-to-reach households. Prior to the implementation, there was no available transport at all, making visits in these remote areas challenging, sometimes impossible. More regular access to a car, however, remained a challenge and was repeatedly mentioned by CHWs as a need for program improvement.

*“Before we were working and walking distances alone with no one, now the work is easier due to their presence and support. Secondly you would find out that now we even visit far places because at least now there is a car”. (*Intervention CHW)

On the other hand, control CHWs reported a dire need of tangible resources to enable them to conduct their work.

*“This programme, right. Firstly, I was going to start providing equipment for them where they will be able to, where they will work, be sure to work efficiently because they have everything. So that you don’t get people not doing what they’re supposed to just because she does not have the essentials to do the job.” (*Control CHW)

### 3. CHWs experiences of supervision

#### 3.1 Supportive supervision leads to an increased sense of accountability through supportive supervision

Intervention CHWs clearly described initial challenges when the study was introduced, where CHWs were apprehensive about the training and the program as a whole. Supervisors report that initially it was very challenging to supervise CHWs who are not actually employed by the organization supervising them. In addition, they reported challenges in supervising CHWs whose experiences of supervision was very different to the approach in the intervention. They stated that these concerns dissipated over time.

*“In the beginning they had a bad attitude, they did not want to listen even in meetings, telling us that they do not want this, do not want the bags that you give us to carry to the community. There was these phone that they said: it’s “police” that you may see who is at work and who is not. In the beginning they had just a book, but now they have all the material”* (Intervention supervisor)

The increased sense of accountability through the intervention became evident.

*“I think supervision is the person who comes to check my work, on how I do it and show me where I’m wrong and teach me where I need to be taught. I mean that person improves my work because he/she gives me the power to do better at work. If I had made a mistake I would then pull up my socks and do my job the right way” (*Intervention CHW)

#### 3.2 Respect and positive supportive relationships critical for supervision

Relationship building between supervisors and CHW emerged as a vital facilitator for enabling supervision. Supervisors described how, as they got to know their group of CHWs over time, their relationships with CHW allowed successful supervision to became possible. CHWs described the importance of supervisors being respectful and supportive in their approach. Supervisors also described the importance of finding a balance between being “a friend” to CHWs and at times, being firm about job responsibilities. Supervision strategies mentioned by supervisors included being respectful and supporting, and that communication is key.

*“It is fun because even them they don’t take themselves as supervisors they just take themselves as CHWs just like us. … if you are talking she would support what you just said and it would be great even in that house, even when you walk with them on the road it is great, you don’t even feel like you are with your supervisor, you are walking with another CHW just like me”* (Intervention CHW)

*“I think that it’s nice to have a supervisor even though we needed to adjust to it since we were working on our own before and there were no targets or paperwork. It’s also beneficial to us because I gained a lot of knowledge and understood my work more and it becomes easier as you have a supervisor checking up on you. The organisation also gives us courage to do more for our communities, cover more areas. Overall my experience was good and beneficial to me and to clients after supervision”* (Intervention CHW)

#### 3.3 Higher intensity of supervision increases sense of support

While CHWs in the intervention arm reported having, with time, built close relationships with the Philani supervisors, CHWs in the control condition described limited supervision. Although all clinics in the study had team leaders based at each clinic at the beginning of the study, several had since left their positions and been replaced by Operational Team Leaders based in a town approximately two hours from the clinic, with little to no access to transport. Supervision therefore mostly consisted of a phone call once a week to collect statistics. Some CHWs reported that they received support from Operational Managers [OMs] at those clinics. The OMs however had limited time for supervision as they were nurses, whose main responsibility was clinic-based clinical care.

*“No, we do not get enough support through the phone because there are a lot of things that we do not know since she is not in our midst. Working over the phone in comparison to working face to face, those are two different things. I have never gone for fieldwork with my supervisor”* (Control CHW)

*“We used to have a team leader and that was nice because she used to equip us when we needed that, but now we have a nurse that we talk to when we need help. But the nurse cannot go for the home visits with us because there is a shortage of nurses already in the clinics”* (Control CHW)

*“I do not feel good about it because this sometimes delays my ability to sort out issues that my clients are facing. If I had a fieldworker supervisor, things would be solved sooner. For example, something that would normally take months to solve you would see that it takes a whole year to solve” (*Control CHW)

## Discussion

This paper describes CHW experiences of training, resource provision and supervision in two arms of a cRCT. Participants described several shortfalls in the government-implemented CHW programme in the study area in these domains. Our findings show that the intervention package provided through this study temporarily (during the intervention) mitigated some of these shortfalls and CHWs reported higher job satisfaction and motivation as a result of the added training, support and supervision.

Our findings suggest that CHWs in the standard government system are operating in isolation and with little support. These findings are concerning considering the recommendation of a supportive system that is required for successful implementation [2, 35]. On the other hand, CHWs receiving supervision and other resources as a part of the cRCT reported that it has had significant positive impact on their motivation and ability to carry out their work effectively. These findings are important for understanding the building blocks of a functional supervision system and how these building blocks can be improved to create a more effective CHW program.

Large scale CHW programs face many challenges [36], and often - once scaled - the effectiveness partly falls away [37]. The focus in many studies is on informational content, messages, dosage and there is a need to better understand the nature of supervision provided, what resources are available, what training is conducted – and how these elements are experienced by CHWs themselves. Given the struggles of the CHWs in the control arm of this study, it appears that important building blocks are neglected or absent. This has been the case in many national CHW programs [37, 38]. The intervention in this study has somewhat mitigated these challenges and lessons learnt from it could be useful for other CHW programs.

From our interviews, it is clear that initial buy-in and engagement for the training that was conducted as part of the cRCT was low. It appears that CHW were required to perform tasks that they did not fully understand or believe in and were not supported to perform their duties. It is encouraging, however, that after the training a new understanding of the value and potential of the intervention was created, seemingly leading to improved confidence, self-efficacy and motivation for CHWs.

It is clear that training plays a major role in CHW program success. The additional training provided in this study improved CHWs knowledge and confidence and there appears to be scope for more research on both the quality of CHW training and on the need for ongoing in-the-field and other trainings [7]. Furthermore, our findings suggest that training needs to be paired with access to equipment, transport and supervision to be fully effective. CHWs in both arms of the cRCT report how the added training made a major difference in their knowledge and motivation. But knowledge without accountability and essential equipment limited the ability of CHWs in the control arm to fully make use of their skills. The added resources (equipment and transport) provided to the intervention CHWs substantially improved their ability to perform their job, which in turn improved their motivation. It is concerning that such an important building block of the CHW program is so neglected in the current system [39]. This needs to be addressed. It is possible that limited access to equipment and resources is linked to financial constraints [40], or a lack of political will or coordination [39].

CHWs in this study report major differences in the way supervision was conducted in the two arms of the trial. Findings suggest that the frequency and approach of the supervision for control CHWs was poor, echoing current evidence [14]. Intervention CHWs report an earlier lack of supervision, whereas during the study they felt more supported. The supervision approach that the CHWs did experience previously appeared to have been more fault-finding/punitive than supportive, which is reported both by CHWs in this study and others [41–45] as being demotivating. There should be a stronger focus on supervisor training [44] and on supervision strategies in designing and implementing CHW programs [13]. Given that both CHWs and supervisors in the intervention arm experience the relationship between them as a main contributing to factor to successful supervision, it is deeply concerning that supervisor posts in the control arm are either not filled, or filled by a supervisor not based in the intervention area and that none of the control CHWs were ever accompanied in the field by their supervisors. This raises the question of whether health facility supervisors [professional nurses] actually are best suited for providing supervision to CHWs. They often work in overburden health facilities with little or no time for additional activities and are usually bound to their facilities [13, 46].

CHW confidence and motivation increased through improved knowledge, skills and support [47, 48] leading to better service delivery and through that, increased program credibility and community uptake. Supervision in the intervention arm of this study and others [52], appears to enhance program credibility in the community, which facilitates program acceptance. Further, the knowledge gained through Philani trainings emerged as a facilitator for community acceptance. Prior to the implementation of the Philani system, CHWs only worked with notebooks, and they describe that as they now have scales, some medication and folders – they are taken more seriously by the community and thus find it easier to gain acceptance, resonating with current literature describing how equipment enhances program credibility [42].

When planning new CHW programs, it is important to keep in mind that these building blocks work together to create a functional system in which the CHW can work effectively. Unless sufficient resources for training, supervision, equipment, and logistic support such as transport, are available – a CHW program may be inefficient. Intervention CHWs report feeling that they have more to offer the community now as they are better trained, have got access to equipment, and are being supported by supervisors. Given the importance of supervisor skills and the relationship-building required for successful supervision, more attention should be given to supervisor training. Involving all stakeholders, including CHWs and supervisors should be considered when designing CHW programs [46], and supervision strategies. Equally important to note is that it took more than 18 months for this intervention to settle, an important finding especially in light of many CHW research programs that are limited in timescale.

Our findings highlight which CHW program domains that are important for CHWs themselves and indicate that adequate resources be allocated to training, supervision, equipment and logistical support such as transport when designing a CHW program. CHWs in the control arm were more motivated after receiving additional training but experienced many challenges in putting their knowledge to use as resources and supervision was missing. The intervention in this study seem to have mitigated some of these challenges by providing additional training, equipment, transport and supportive, in-the-field supervision. In a low resource setting like the rural Eastern Cape, CHWs deliver a valuable service to their communities and need to be supported through a functional supervision system, especially in the light of an emerging focus on CHW rights and needs [48]. Our findings show that the package delivered through the Philani model can improve CHW motivation and work performance, which in turn improves community uptake and health outcomes.

## Limitations

It is important to note the limitations of this study. Firstly, the CHWs interviewed in this sub-study were from a relatively small pool of Department of Health-employed CHWs taking part in a larger cRCT. This may have caused concerns about confidentially and furthermore it may have had an impact on the level of honesty in the interviews, particularly with critical feedback. We do not believe this was the case in this study, as various measures were put in place to ensure confidentiality, as described, and the feedback of DoH support was quite critical. Furthermore, the interviewer was not previously known to the CHWs and was completely independent of the EC DoH, which we believe was an advantage. Secondly, we did not have the capacity to conduct individual interviews with the full sample, and chose to randomly select CHWs for individual interviews. Some CHWs started as CHWs later than others, which may have affected their experiences. Thirdly, the first author has been involved in the Philani program, with both intervention and evaluation, which may have influenced how the data was viewed. This potential risk was mitigated by having an external interviewer conduct the interviews and having a second researcher analyse sections of the data.

## Conclusion

For CHW to be able to work effectively, a functional and supportive supervisory system is critical. The integration a supervisory model consisting of additional training, logistical support, and support and supervision in the field, appears to have improved CHW motivation and performance in this study. Our findings highlight the importance of allocating sufficient resources for, equipment, transport, training and in-the-field supervision when designing and implementing CHW programs. CHWs have an important role in providing primary health care - particularly in rural areas - and more emphasis needs to be put on key programmatic building blocks such as training, equipment and transport and particularly on effective and supportive supervision.
